# Illness anxiety disorder

**DOI:** 10.1192/j.eurpsy.2021.684

**Published:** 2021-08-13

**Authors:** P. Hervias Higueras, S. García Jorge, J. Correas Lauffer

**Affiliations:** Psiquiatría, Hospital Universitario del Henares, Coslada, Madrid., Spain

**Keywords:** illness anxiety disorder, somatic symptom and related disorders, hypochondria

## Abstract

**Introduction:**

The diagnosis of hypochondria has disappeared in the new classification of mental illness. About 25% of patients who were diagnosed with hypochondria now fall into the category illness anxiety disorder. This disorder constitutes a new diagnostic category in DSM5 and is included within the somatic symptom and related disorders.

**Objectives:**

We propose to carry out a bibliographic review off the new diagnostic category of illness anxiety disorder.

**Methods:**

We present the clinical case of a 27-year-old man in the context of the Covid19 pandemic.

**Results:**

The illness anxiety disorder is characterized by being concerned about having or acquiring a serious illness. Somatic symptoms are not present, but if they are, they are of mild intensity. The level of concern is excessive or disproportionate if there is any disease or if there is a high risk of developing it. There is a high level of health anxiety and the individual is easily alarmed by personal health status. It is a disorder that tends to be chronic and recurrent. The exact comorbidity is still unknown. However, it is important to keep in mind that hypochondria concurs with anxiety disorders and depressive disorders. Treatment is based on the cognitive restructuring of bodily symptoms. In addition, exposure therapy and acceptance and commitment therapy are also effective. Regarding pharmacological treatment, SSRIs are useful in relation with comorbidity.

**Conclusions:**

Illness anxiety disorder is characterized by significant attention to somatic concerns in medical places, making it very useful for primary care professionals.

